# Achieving well-informed decision-making in drug discovery: a comprehensive calibration study using neural network-based structure-activity models

**DOI:** 10.1186/s13321-025-00964-y

**Published:** 2025-03-05

**Authors:** Hannah Rosa Friesacher, Ola Engkvist, Lewis Mervin, Yves Moreau, Adam Arany

**Affiliations:** 1 Department of Electrical Engineering (ESAT), STADIUS Center for Dynamical Systems, Signal Processing and Data Analytics, KU Leuven, Leuven, 3000 Belgium; 2 Department of Computer Science and Engineering, Chalmers University of Technology, Gothenburg, 412 96 Sweden; 3 Molecular AI, Discovery Sciences, R&D, AstraZeneca Gothenburg, Gothenburg, 431 83 Sweden; 4 Molecular AI, Discovery Sciences, R&D, AstraZeneca Cambridge, Cambridge, CB2 0AA UK

**Keywords:** Drug discovery, Deep learning, QSAR, Uncertainty estimation, Probability calibration, Bayesian neural network, Hamiltonian monte carlo sampling

## Abstract

In the drug discovery process, where experiments can be costly and time-consuming, computational models that predict drug-target interactions are valuable tools to accelerate the development of new therapeutic agents. Estimating the uncertainty inherent in these neural network predictions provides valuable information that facilitates optimal decision-making when risk assessment is crucial. However, such models can be poorly calibrated, which results in unreliable uncertainty estimates that do not reflect the true predictive uncertainty. In this study, we compare different metrics, including accuracy and calibration scores, used for model hyperparameter tuning to investigate which model selection strategy achieves well-calibrated models. Furthermore, we propose to use a computationally efficient Bayesian uncertainty estimation method named HMC Bayesian Last Layer (HBLL), which generates Hamiltonian Monte Carlo (HMC) trajectories to obtain samples for the parameters of a Bayesian logistic regression fitted to the hidden layer of the baseline neural network. We report that this approach improves model calibration and achieves the performance of common uncertainty quantification methods by combining the benefits of uncertainty estimation and probability calibration methods. Finally, we show that combining post hoc calibration method with well-performing uncertainty quantification approaches can boost model accuracy and calibration.

## Scientific contribution

In this work we provide a comprehensive probability calibration study using neural networks for drug-target interaction predictions. We report a significant effect of the hyperparameter selection strategy, as well as uncertainty estimation and probability calibration approaches on the reliability of uncertainty estimates, which is crucial for an efficient drug discovery process.

## Introduction

The development of safe and effective drugs is a challenging task associated with high development costs, a high risk of adverse effects or lack of efficacy, which can lead to the failure of a drug candidate or to long approval processes until a drug can be brought to the market [1, 2]. Machine learning models have emerged as a valuable tool, revolutionizing the drug discovery and development process by shifting to a more time- and resource-efficient pipeline [3–5].

As a consequence of the increasing availability of computational resources and data, recent machine learning models perform well in prediction tasks, which is reflected in high accuracy scores and low classification errors. Estimating the uncertainty inherent in such a prediction can provide a valuable source of information in various applications besides drug design [6–12]. In drug discovery, accurate uncertainty estimates can be leveraged to improve decisions about which candidates to pursue across a candidate portfolio.

The reliability of uncertainty estimates is crucial to guarantee the trustworthiness of machine learning models. This is particularly important for high-stakes decision processes like the drug discovery pipeline where experiments can be costly and poor decisions inevitably lead to an increase in required time and resources. Even when prediction accuracy is good, neural networks often fail to give realistic estimates of how uncertain they are about a prediction. These models are called poorly calibrated, which implies that the model’s confidence does not reflect the true probability of making a prediction error. In this context, confidence is defined as the degree to which the model thinks that the prediction is correct. In the case of binary classification, a prediction of 0.5 implies minimum confidence, while the confidence increases with increasing shifts toward the extremes of the probability interval. An overconfident model is too confident in its predictions, which are skewed toward the extremes of the probability range. In contrast, underconfident models generate probabilities that cluster too closely around 0.5, reflecting higher uncertainty. In general, well-calibrated models refer to models whose probabilistic predictions correspond to the true likelihood that an event occurs. For example, if a compound is predicted to be active with a 70% probability, then about 70% of the molecules given that prediction will be active if the model is well-calibrated. Under and overconfidence can be determined by calibration errors (CE) measuring the error between the probabilistic prediction of a classifier and the expected positive rate given the prediction.

Predictive uncertainty can come from various sources. While many different categorizations of these sources can be found in literature, a common one is the distinction between aleatoric and epistemic uncertainty [13, 14]. Aleatoric or data uncertainty is the uncertainty related to data and data acquisition, including systematic and unsystematic errors, such as measurement errors. Aleatoric uncertainty is also often called irreducible uncertainty, as it cannot be decreased by adding more data samples to the current model. In contrast, epistemic, or model uncertainty can be reduced by adding knowledge. Epistemic uncertainty can have several causes, including model overfitting or distribution shifts between training and test data.

In classification, the model output is usually a probability-like score, reflecting the uncertainty of a prediction, if the network is well-calibrated. The predictive uncertainty should summarize the total uncertainty associated with the prediction, considering all sources of uncertainty. However, these probabilities have been reported to diverge from their ground truth preventing a reliable risk assessment [15, 16]. In 2017, Guo et al. [15] drew attention to the inability of modern neural networks to estimate uncertainties of predictions correctly. They reported that despite their high accuracy, large neural networks are poorly calibrated, resulting in inaccurate probability estimates.

In their paper, Guo and his colleagues linked poor probability calibration to model overfitting, leading to increased probabilistic errors rather than affecting the model’s ability to correctly classify test instances. Furthermore, they concluded that model calibration and model accuracy are also likely to be optimized by different hyperparameter (HP) settings [15]. While Guo et al. [15] proposes that the growing size of modern neural networks contributes to poor probability calibration, Minderer et al. [17] found that poor calibration is more related to the model family used (e.g. MLP, ResNet, CNN) than model size. Wang et al. [19] list three major factors diminishing the probability calibration of a model, including large model size and over-parametrization of models, lack of model regularization and data quality and quantity, as well as imbalanced label distribution in classification. In addition, the distribution of training and test data was reported to impact model calibration. Current neural networks are often overconfident so probability calibration deteriorates with increasing distribution shift [17, 20].

As many factors contributing to poor probability calibration cannot be mitigated in real-world scenarios, it is crucial to identify methods capable of addressing these challenges and generating reliable uncertainty estimates. This is particularly important in drug discovery when developing new therapeutic agents, which requires exploring the chemical space by shifting the focus during inference to chemical structures unknown to the model. So far, there is no widespread agreement in the literature on which methods are most successful for achieving calibrated probabilities. Several papers exist that study the quality of uncertainty estimation in models used in various drug discovery applications. These include the application of various uncertainty estimation approaches, including evidential learning [21–23], distance-based methods[18, 24], Gaussian processes [23, 25], Bayesian and ensemble-based techniques [18, 23, 24, 26–29], and conformal predictors [30]. Furthermore, post hoc recalibration techniques have been assessed in classification [16] and regression tasks [31].

While all of these papers provide significant contributions to identify approaches that obtain high-quality uncertainty estimates, there is, to our knowledge, no study investigating the impact of different HP optimization metrics on the calibration properties of bioactivity prediction models. Our study aims to close this gap by contributing an analysis of how to train binary classification models when striving for good uncertainty estimates. Furthermore, we evaluate and compare various uncertainty estimation approaches based on the quality of their uncertainty estimates. We propose a limited computational complexity Bayesian approach, which allows the retrieval of samples from the posterior distribution of the last layer weights. While similar methods have been evaluated using variational approximation approaches in other fields [32–36], we use a Hamiltonian Monte Carlo (HMC) approach for Bayesian approximation for drug-target interaction predictions. Furthermore, we compare this Bayesian approach to the uncalibrated baseline model, two common uncertainty quantification, and one probability calibration method. Finally, we investigate if combining the post hoc calibration approach Platt scaling with other uncertainty quantification methods benefits model calibration. In this work, the probability calibration of classification models is evaluated by assessing different metrics, including calibration errors and the Brier score, that quantify the quality of uncertainty estimates. Detailed mathematical definition of calibration error is provided in the Methods section and in the Appendix.

In conclusion, this paper focuses on exploring the effects of different model selection strategies and approaches for uncertainty estimation and probability calibration on the quality of uncertainty estimates using drug-target interaction modeling.

### Related work and background

#### Post Hoc calibration methods

In this study, post hoc calibration methods refer to approaches that correct probabilities by using a calibration dataset. This dataset is used to fit a calibrating function to the scores of a classifier after the training of the neural network has been completed. These methods do not provide uncertainty estimation of the parameters. However, they provide a first-order uncertainty estimation of the binary prediction [37].

*Platt scaling.* Since 1999, Platt scaling [38] has been widely used for calibrating probabilities [15, 16, 19]. It is a parametric calibration method that fits a logistic regression model to the logits of the predictions of a classifier to counteract over- or underconfident model predictions. Usually, a separate dataset, called calibration dataset, is used for this calibration step. Since Platt scaling is a post hoc calibration method, it is versatile and can be used in combination with other uncertainty quantification techniques, including Bayesian approaches.

#### Train-time uncertainty quantification methods

In contrast to post hoc calibration methods, the uncertainty quantification approaches discussed in this section estimate uncertainty during training. In our work, we define train-time uncertainty estimation approaches as methods that do not need a separate calibration set for correcting the predicted probabilities. The main idea of these techniques is to provide uncertainty of the model parameters by treating the model parameters as random variables with associated probability distributions. Bayes’ theorem allows access to these posterior distributions $p(\theta |D)$ over model parameters $\theta$. Subsequently, a posterior distribution of the predicted label corresponding to the test instance *x* can be derived by marginalizing over $\theta$:

1 $$\begin{aligned} p(y|x, D) = \int _{\theta } p(y|x, \theta )p(\theta |D) \,d\theta . \end{aligned}$$

The Bayesian paradigm is used by Bayesian neural networks, which obtain probability distributions for the network parameters. Since they consider many possible model solutions, they account for uncertainty in the model during inference. However, the model posterior distributions are usually complex, and their analytical form is often not available because of intractable marginal likelihood terms needed for exact Bayesian inference. The majority of the uncertainty quantification approaches are Bayesian or apply heuristics motivated by Bayesian statistical principles. These include various sampling-based approaches that draw samples $\theta _i \sim p(\theta |D)$ from this complex posterior distribution. An uncertainty estimate for a test instance can be obtained by averaging over the samples $p(y|x, D) \approx \frac{1}{M}\sum _{m = 0}^{M} p(y|x, \theta _m)$. The following sections provide a short introduction to the uncertainty quantification methods used in this study. In addition, an overview of the approaches is also provided in Fig. 3.

*Monte Carlo dropout.* In the context of training Bayesian neural networks, Monte Carlo (MC) dropout can be regarded as an approximation to Bayesian inference [39]. In MC dropout, parameter samples are retrieved by passing the input data through the neural network multiple times. Stochasticity is introduced by applying dropout during inference. During each forward pass through the neural network, a new set of randomly selected neurons is set to zero. The mathematical details on forward passes through neural networks is described in Section F.1. This approach resembles the application of dropout during model training [40]. Subsequently, the samples are averaged to obtain an uncertainty estimate for a test instance, as described earlier in this section. Since for MC dropout the training of only one model is necessary, this calibration method is efficient in terms of computational cost and time, as shown in Section D in the Appendix. Due to its simplicity and efficiency, MC dropout has been extensively studied for uncertainty quantification in cheminformatics applications [24, 29, 41].

*Deep ensembles.* Another approach that has been shown to produce high-quality probability estimates is the generation of deep ensembles [29, 42]. Deep ensembles have also demonstrated greater robustness to data shifts compared to other widely used uncertainty quantification methods [20].

For uncertainty quantification with deep ensembles, multiple base estimators are trained, starting from different weight initializations of the network. To retrieve a single probability estimate, the predictions of the different base estimators are averaged, as described at the beginning of Section 1.1.2. It is assumed that because of the strong non-convex nature of the error landscape, most of these models reach different local minima, as reported by Fort et al. [43]. It is, therefore, expected that such sets of base estimators represent the most important regions of the posterior.

Deep ensembles are easy to implement. They can however be computationally expensive as they involve the generation of multiple models. A comparison of the the different training and evaluation times of the models is listed in Section D.

*HMC Bayesian last layer*. Partial Bayesian neural networks, which apply Bayesian approximation solely to the final layer of a neural network, have already been introduced in the literature [32–36]. Harrison et al. recently showed that variational Bayesian last layer (BLL) models enhance the calibration and accuracy of baseline models in classification and regression tasks [44]. Note that all of these works use variational approximation approaches while we apply HMC to retrieve samples from the posterior of the last layer weights.

Hamiltonian Monte Carlo (HMC) is a Markov Chain Monte Carlo (MCMC) method, which allows drawing samples directly from the posterior distribution of the parameters [45]. MCMC methods generate samples by constructing a Markov chain in which the proposal distribution of the next sample depends on the current sample. When comparing it to other MCMC methods that use a random walk approach, HMC stands out because of its ability to propose new samples in an informed way. The HMC sampler uses Hamiltonian dynamics to efficiently move through the negative log space of the unnormalized posterior by following Hamiltonian trajectories. Simply put, the sampling procedure can be intuitively imagined as a particle sliding along the space. This particle is stopped after some time to record the current state as a sample of the Markov Chain. The particle moves along specific trajectories obtained by numerically solving Hamilton’s equation. To account for accumulated error, an additional Metropolis-Hastings step is required after drawing the sample, in which erroneous samples can be rejected. For a more detailed explanation of the mathematical and physical details of HMC we refer to [45, 46].

Because its informed approach to proposing new samples, HMC is generally better at generating well-mixing chains than methods using random walk techniques. Furthermore, the mixing ability of the chain will depend on the length of the trajectory determined by the number of steps *L* and the stepsize $\epsilon$. If a chain is mixing poorly, the chain will get stuck in one area of the negative log probability space, resulting in highly correlated samples. If this is the case, the trajectory can be lengthened by either increasing $\epsilon$ or *L*. However, these HPs need to be tuned carefully, since high $\epsilon$ can lead to increased rejection of the proposed samples because of larger accumulated error, and the increase of *L* is often connected to problematic computational costs. Furthermore, tuning the mass matrix *M* can support the generation of efficient HMC samplers by de-correlating the parameter space.

Because of its high computational demand, the application of HMC to a full Bayesian neural network is challenging. In 2021, Izmailov et al. [47] generated truly Bayesian neural networks by training modern architectures using full-batch HMC. Despite giving highly interesting insights into the nature of Bayesian neural networks, the authors concluded that HMC is an impractical method because of the high computational demand. In our work, we propose to use HMC in a computationally feasible way by sampling only from the weight posterior of the last layer of the neural networks. Consistent with the terminology used in the existing literature [32, 33, 36, 44], we refer to the method as HMC Bayesian Last Layer (HBLL). The algorithm of HBLL is reported in Section G.

## Methods

### Datasets

*Extraction of target specific data from ChEMBL.* Target-specific bioactivity data was extracted from the ChEMBL database (version: 29) [48]. To generate single-task models, compound activities from three different targets, namely Monoamine oxidase A (MAO-A), Cytochrome P450 3A4 (CYP3A4), and hERG, were extracted. The targets were chosen to represent common optimization problems in the drug discovery process. CYP3A4 plays an important role in drug-drug interactions and the metabolization of compounds [49, 50]. Furthermore, the inhibition of hERG can lead to severe cardiac side effects [51]. Hence, detecting interactions with these proteins is essential to evaluate the pharmacokinetic and toxicity profile of drug candidates. MAO-A, on the other hand, was chosen as representative for a class of targets that are typically modulated to achieve a desired biochemical effect. MAO-A is modulated by therapeutic agents treating behavioral and neurological disorders. Table 1 summarizes the properties of the used target data. Bioactivities were converted to pIC50, and thresholds for assigning bioactivity labels were chosen for each target, respectively. For MAO-A and CYP3A4, a pIC50 value of 5.5 was chosen as threshold, which resulted in active ratios of approximately 25% for both targets. To investigate if our conclusions were also valid for smaller active ratios, we chose a stricter threshold of 6.5 pIC50 for the remaining target hERG leading to an active ratio of 7% in this dataset. Extended connectivity fingerprints (ECFPs) (size = 32k, radius = 3), were obtained using RDKit Version 2022.03.2[52] which were used as model inputs. More advanced methods are available for molecular representations and model architectures, including graph neural networks for handling molecular graphs and language models for processing SMILES representations. However, since the aim of this method is to identify modeling approaches that produce high-quality uncertainty estimates rather than find the overall best model, we chose the simple ECFP representation. Furthermore, the combination of ECFP with multilayer perceptions, as used in this study, has been shown to outperform other deep learning approaches [53].

**Table 1:** Assay data used in this study. Details for the assay data extracted from the ChEMBL dataset are reported. Data from three assays of varying sizes and positive ratios were extracted

ChEMBL - IDs	Target	#Results	Active Ratio	pIC50 Threshold
CHEMBL340	Cytochrome P450 3A4	7619	0.252658	5.5
CHEMBL1951	Monoamine oxidase A	2917	0.259170	5.5
CHEMBL240	hERG	9558	0.079828	6.5

**Fig. 1:**
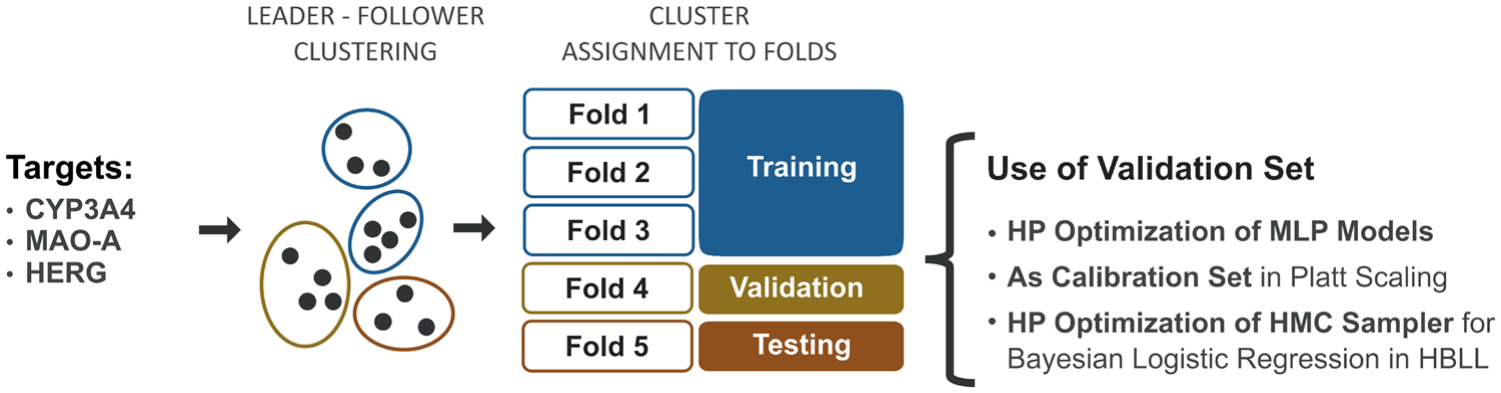
Overview of the dataset generation. The chemical structures were extracted from ChEMBL, and subsequently filtered and clustered. The clusters were assigned to five folds, which were used to set up a training, validation, and test fold. The training folds were used for MLP training. The validation set was used for HP tuning, as well as for fitting the logistic regression models for the Platt-scaled models, and to choose the prior for HMC Bayesian last layer model (HBLL), respectively

*Fold generation via clustering.* The data was split into five different folds to enable cross-validation. In cross-validation, data is excluded from the training process, which is used to validate and evaluate the model after training. This allows the assessment of the model’s generalizing abilities by evaluating its performance on unseen data. In our study, we used three folds for model training, one for validation, and one for testing. Fig. 1 illustrates the generation and use of the dataset splits. The validation fold used for the model’s quality assessment during HP tuning and for early stopping was excluded from the training dataset. To obtain the folds, we used the procedure of fold generation described in detail in Simm et al. [54]. In short, Tanimoto similarity computed on the above-described ECFP features is used to measure the chemical similarity of the compound, which is then used to assign the compounds to clusters. Next, the entire clusters containing similar compounds were randomly assigned to folds. This procedure ensures that training and test datasets consist of compounds from divergent chemical space, mimicking the real-world scenario, in which the model is used to predict bioactivities for chemically unfamiliar compounds. Testing the model on compounds to those on which it was trained would result in overoptimistic results during model performance assessment.

### Single-Task model generation

**Fig. 2:**
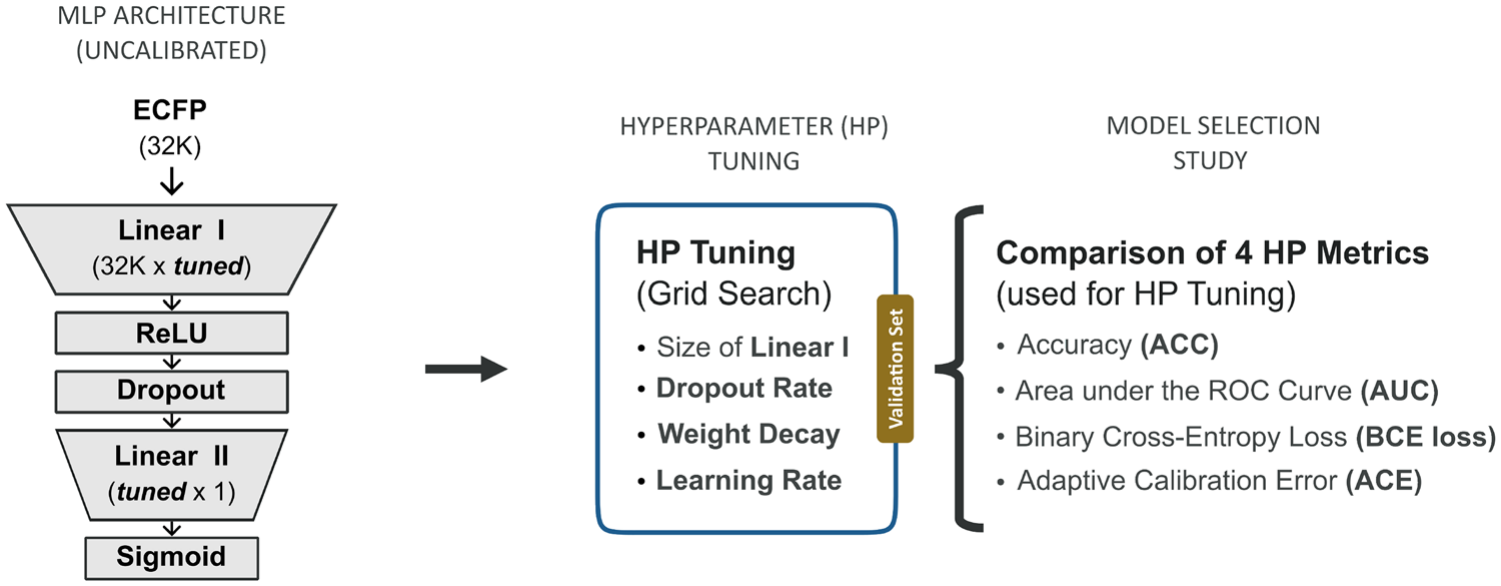
Overview of the architecture of the MLP baseline model and the HP tuning workflow. The size of the hidden layer and the dropout rate, as well as the weight decay and learning rate used during training, were tuned in a grid search using a validation dataset. Four different HP optimization metrics (HP metrics) were used, and the performances of the respective models were compared in a model selection study

*Model architecture.* In this study, we adopt the modeling framework SparseChem, proposed by Arany et al. [55] which has been used by multiple industry stakeholders [56]. Single-task feed-forward multilayer perceptrons (MLP) were generated and used as the baseline for binary classification tasks. The mathematical background for training and predicting with MLPs is outlined in Section F in the Appendix. Subsequently, the baseline models were extended to compare different uncertainty estimation and probability calibration approaches. Figure 2 illustrates the architecture of the baseline MLPs and the HP optimization workflow. The baseline MLP is comprised of two layers, with a ReLU function and a dropout layer in between. Probability-like scores were obtained by applying a sigmoid function. The size of the hidden layer and the dropout rate were tuned in a grid search as described in the next section. Deeper MLPs with 2 to 4 additional layers were tested to evaluate whether they could enhance the performance. The results of this experiment show no significant improvements in using deeper MLPs, as shown in Section B.1 in the Appendix. With regard to the HBLL model, the performance of HMC samplers in the last layer should be comparable independently of the depth of the network. Sommer et al. [57] demonstrated that the mixing of HMC samplers improved in deeper layers of the network, while it was harder in the first and last [57]. Considering these findings, we opted for the shallow MLP structure, as described above. Models were implemented and trained using PyTorch Version 2.1.0 [58]. The open-source hamiltorch package [59] was used to generate the HMC Bayesian last layer (HBLL) models using a HMC sampler [60].

*Model tuning.* The baseline models were trained using the binary cross-entropy (BCE) loss. A validation dataset was used for early stopping during training and to optimize the HPs of the models. An exhaustive grid search in parameter space was performed to tune the size of the hidden layer, and the dropout rate of the model, as well as the learning rate and the weight decay used during model training as shown in Fig. 2. The space that was considered during HP tuning is reported in Table 8 in the Appendix. For each HP setting, the HP metric was averaged over ten model repeats, which were initialized randomly, to ensure repeatability. Binary cross entropy loss (BCE loss), adaptive calibration error (ACE), accuracy (ACC), and area under the ROC Curve (AUC) were used for model selection to assess the impact on probability calibration.

*Model evaluation.* All models included in our work predict scores between 0 and 1, indicating the probability that a compound is active on the respective target. Note that while the train-time uncertainty methods considered in this work account for epistemic uncertainty in their estimates, the baseline MLP and Platt scaling approach also provide probability-like scores. By incorporating uncertainty in the model parameters, we access an additional layer of uncertainty in the prediction, which can be referred to as second-order estimation. This type of estimation quantifies uncertainty over the parameter of the Bernoulli distribution corresponding to the probabilistic prediction [37]. In contrast, the baseline MLP and Platt scaling methods can be seen as first-order uncertainty estimation approaches.

All performance metrics were calculated from predictions on a test dataset for each model and HP tuning strategy or probability calibration method. AUC ($\uparrow$) scores were obtained to assess the model’s ability to correctly classify samples. In addition, we assessed whether the generated models were capable of producing calibrated probability predictions. We used the BCE loss ($\downarrow$), the Brier score (BS) ($\downarrow$), and two types of calibration errors (CEs) ($\downarrow$) to measure the probability calibration of the models.

In the context of probability calibration, it is important to distinguish between proper and improper scores. While a good proper score implies an overall good model, this is not necessarily the case with improper scores. Using the properties of Bregman divergence, proper scores can be decomposed into two parts: one related to calibration error and one related to the predictive performance of the model. The exact definition of proper scores and the mathematical details of the decomposition can be found in Section E in the Appendix. Proper scores include the BCE loss and the Brier score. The Brier score (BS) [61] measures the performance of a model by obtaining the mean squared error between the predicted probabilities *f*(*x*) and the true labels *y*:

2 $$\begin{aligned} BS = \frac{1}{N} \sum _{n=1}^{N}(f(x) - y)^2. \end{aligned}$$

As stated above, the BS can be decomposed into a calibration and refinement term. The decomposition of the Brier score is demonstrated in Section E.3 in the Appendix. Similar decompositions can be performed for other proper scores, like the BCE loss [62, 63].

In contrast, CEs are improper scores that do not consider the refinement but measure only the calibration error instead. The most frequently used calibration error is the $L_1$ distance between the predictions *f*(*x*) and the expected outcomes given the predictions $\mathbb {E}[y|f(x)]$

3 $$\begin{aligned} CE = \mathbb {E}[|\mathbb {E}[y|f(x)]-f(x)|] \end{aligned}$$

where $\mathbb {E}[y|f(x)]$ denotes the accuracy (*acc*) and f(x) the confidence (*conf*) of the predictions. On an intuitive level, the accuracy represents the expected fraction of positives given the prediction. Since this expectation cannot be calculated, a binning strategy is needed, which allows the expectation to be taken over the predictions assigned to a bin. Both CE types considered in this study estimate the true calibration error by discretizing the probability interval of the predictions into bins $b = \{1,2,.., B\}$ and taking a weighted average of the errors over all bins [64, 65]. Thus, the calibration error is calculated as following

4 $$\begin{aligned} CE = \frac{1}{N} \sum _{b=1}^{B} n_b \left| acc(b) - conf(b)\right| . \end{aligned}$$

in which $n_b$ stands for the number of instances in bin *b*, *N* for the total number of predictions, *acc*(*b*) for the accuracy and *conf*(*b*) for the confidence in bin *b*. The expected calibration error (ECE) is commonly used in literature to assess if a model is calibrated. In addition, we also used the adaptive calibration error (ACE), which has some desirable properties making it more robust towards skewed distributions of the predictions, as described below. The ECE and ACE differ only in the way in which the bins are formed. While for the ECE the probability interval is divided into equally-spaced bins of a fixed width, the ACE forms bins with the same number of samples in each bin [65]. In general, the ACE is considered more robust since the constant bin size prevents samples from contributing more to the error than others. In contrast, this behavior can be detected in the ECE as a result of the fixed binning leading to differently populated bins and an increased variance of the error estimate in bins with fewer samples.

It is important to highlight that because these CEs are improper scoring rules [66], predictions with zero calibration error are not necessarily good predictions. A model that always predicts the overall ratio of class instances in the dataset will be perfectly calibrated, despite its poor accuracy. Nevertheless, these scores are useful in assessing and comparing the probability calibration of models, which aligns with the goals of our paper. Consequently, we will employ these scores alongside proper scoring rules to evaluate both the calibration and overall performance of the models.

### Experiments

For the sake of repeatability and estimation of the standard deviation of the predictions, we generated ten model repeats with random seeds for each model type and averaged the resulting repeats. Note that for computational reasons, only five repeats were generated for ensemble models, resulting in 250 training sessions per target. The statistical significance of the best results was tested in each experiment by performing a two-sided *t*-test with a threshold of *p* = 0.05. Paired *t*-tests were used when comparing the baseline model with modifications of this specific model or between these modifications, including MLP plus Platt scaling (MLP + P), MC dropout (MLP-D), HMC Bayesian last layer (HBLL) and HBLL plus Platt scaling (HBLL + P). In all other cases, an unpaired *t*-test was used.

*Model selection study.* Since it is assumed that model overfitting affects probability calibration, we assessed the impact of different HP optimization metrics (HP metrics) on model calibration. To do so, we compared the calibration errors of models with HPs either maximizing accuracy (ACC) or the AUC value or minimizing the BCE loss or the ACE. We assessed how the AUC, the CEs, and the BS were affected by varying HP metrics. Furthermore, we compared if the results of this analysis vary across targets.

**Fig. 3:**
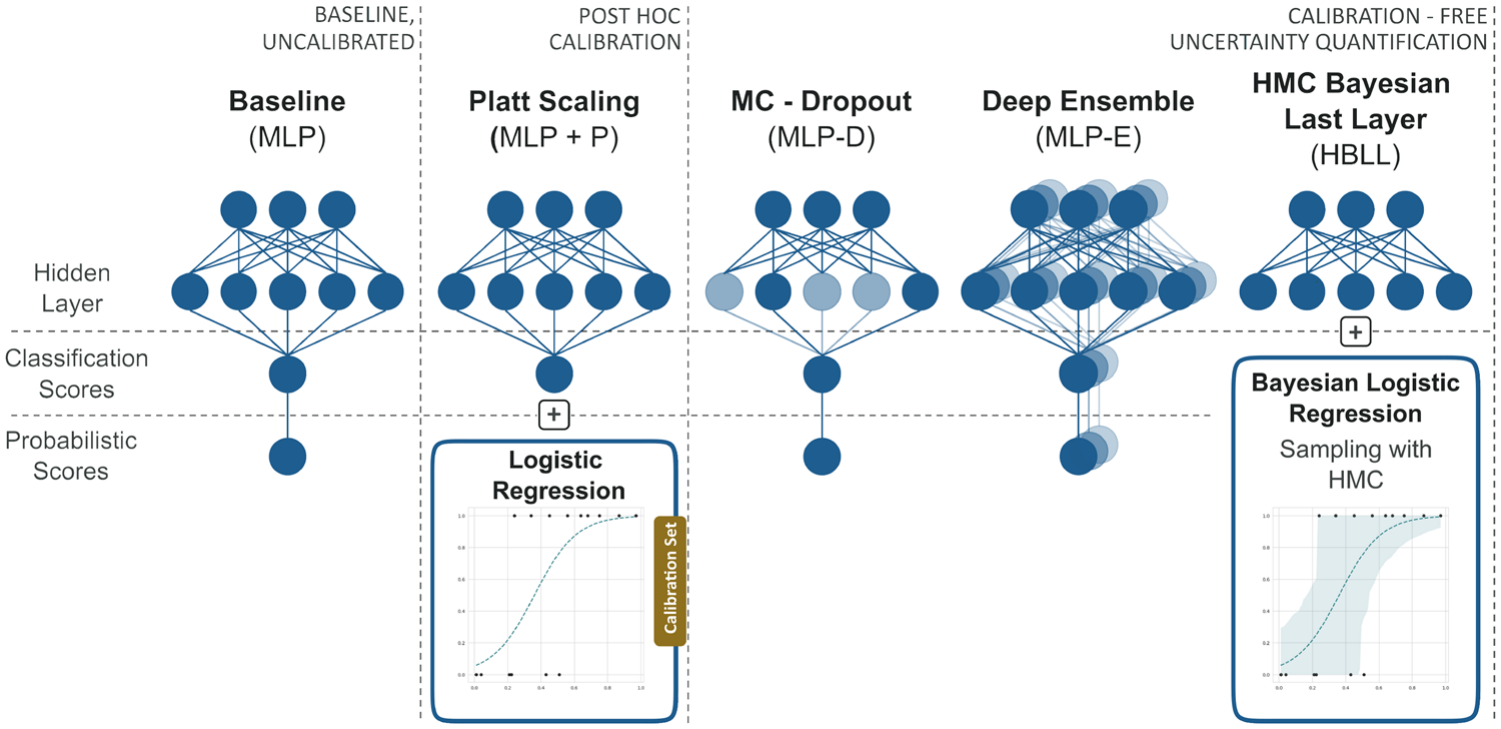
Overview of model architectures assessed in the model calibration study. The baseline model (MLP) was compared to the post hoc calibration method Platt scaling (MLP + P) and the Bayesian approaches MC dropout (MLP-D) and deep ensembles (MLP-E). Furthermore, the proposed Bayesian approach HMC Bayesian last layer (HBLL) was included in the analysis. The models were trained on the training dataset. For the post hoc calibration approach, the validation dataset was used to fit the logistic regression model

*Model calibration study.* We studied the ability of three uncertainty estimation techniques and one post hoc calibration approach to generate predictions with smaller calibration errors than the baseline, indicating better uncertainty estimates. Fig. 3 gives an overview of the methods assessed in this experiment.

In all cases, we build on an uncalibrated multilayer perception baseline model, which we refer to as MLP or baseline model in this paper. For the post hoc calibration method, Platt scaling, the validation dataset was used to fit a logistic regression to the generated scores. In the following sections, we will refer to this model as MLP + P. Moreover, we assessed two uncertainty estimation approaches: ensemble models (MLP-E) and MC dropout (MLP-D). For the generation of MLP-E models, 50 base estimators were trained with random initialization, whereas for the MLP-D approach, 100 predictions were generated using dropout during the forward passes. In both approaches, the predictions were averaged to obtain a prediction for a test instance. Finally, we used our proposed method HMC Bayesian Last Layer (HBLL), by removing the last layer of the baseline MLP and replacing it by a Bayesian logistic regression model.

The parameters for the logistic regression model were sampled from their true posterior distribution using HMC. To generate the HBLL models, a vanilla HMC approach with one sampling chain was chosen with 500 burn-in and 1000 samples. The trajectory length and integration timestep was chosen based on preliminary runs and fixed to $L=1200$, $\epsilon =0.01$. No additional adaptation method, such as NUTS, was used. The inverse mass matrix was set to the Hessian corresponding to the Maximum A Posteriori (MAP) parameter estimate of the logistic regression model. Note that similarly to MLP-E and MLP-D the training of the HBLL model is carried out on the training set and does not use a calibration set. The validation set was used to tune the precision of the Gaussian prior of the model parameters. The parameter space considered during tuning the precision is reported in Table 9 in the Appendix. The selection of this single scalar parameter has an analogous effect as regularization and positions the method between the two main types of methods discussed so far. Again, CEs, BS, and AUC scores were used to compare the performance across model architectures and targets.

**Fig. 4:**
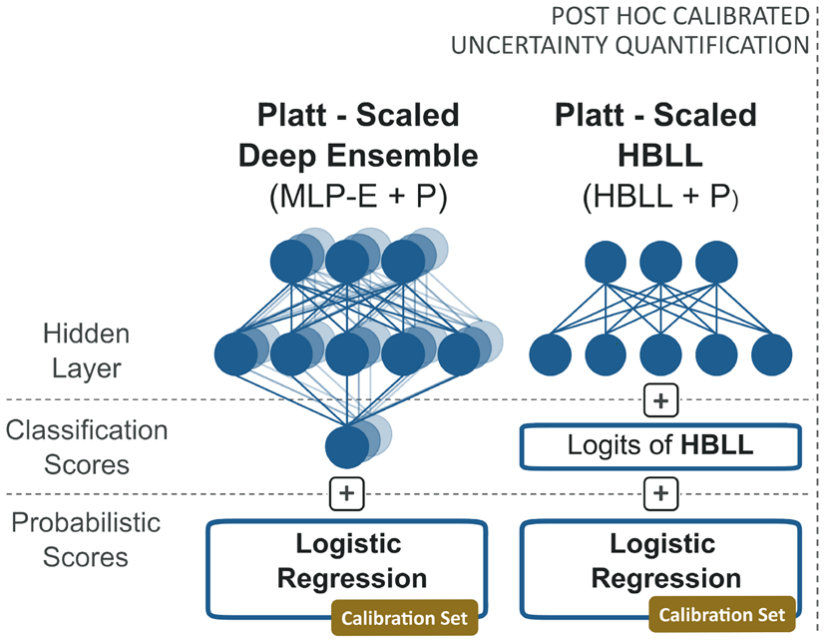
Architecture of the uncertainty estimation approaches combined with Platt scaling. The architectures of MLP-E + P and HBLL + P are shown. For generating Platt-scaled uncertainty quantification methods, a sigmoid was fit to the logits of the deep ensemble (MLP-E) and HMC Bayesian last layer (HBLL) model. The validation dataset was used as calibration set

As a second step, post hoc calibration and uncertainty quantification methods were combined to assess whether the model’s probability calibration would benefit from first quantifying the uncertainty inherent in the predictions and subsequently calibrating the uncertainty estimates. The architecture of the combined models is illustrated in Fig. 4. We applied Platt scaling to the MLP-E and HBLL model, by fitting a sigmoid function to the logit scores of the predictions, which resulted in the Platt-scaled uncertainty quantification models MLP-E + P and HBLL + P. Since Platt scaling does not affect the AUC scores of the predictions, only CEs and BS were calculated to compare the models’ performance with their platt-scaled counterparts.

## Results and discussion

Reliable uncertainty estimates are crucial for assessing the costs and benefits of experiments in the drug discovery process. They can support the identification of compounds that are more likely to be active against a target of interest and on which further experimental analysis should be focused. In the following sections, we evaluate several HP tuning strategies to assess their ability to produce calibrated models. Furthermore, we compare different performance metrics, such as the CEs, to assess the ability of various probability calibration and uncertainty estimation approaches to generate high-quality uncertainty estimates. These experiments will identify practices that can generate well-calibrated machine learning models.

### Model selection study

We investigated the calibration of baseline models with four different HP settings, each of which was selected to optimize ACC, AUC, BCE loss, and ACE.

**Fig. 5:**
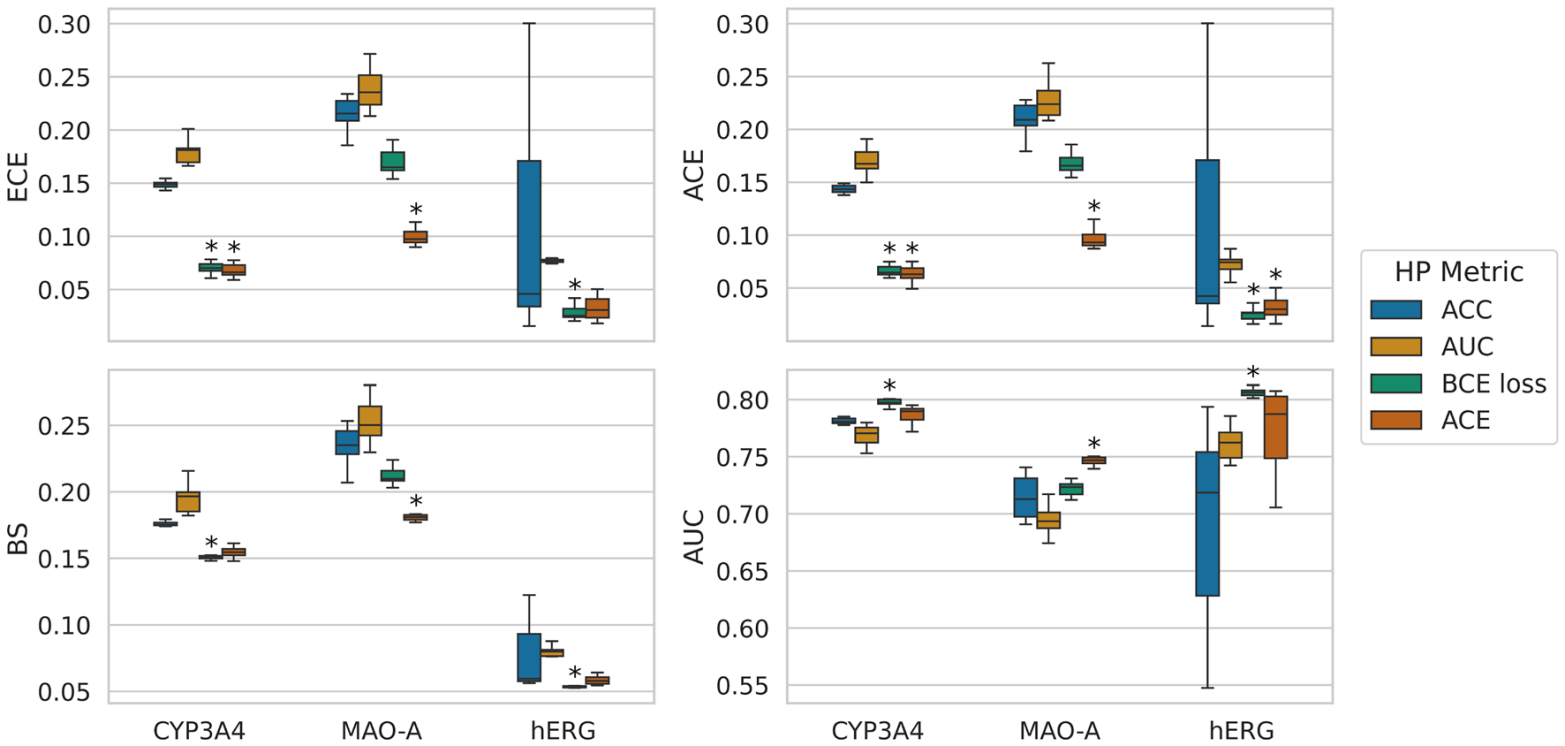
Results of the model selection study for all targets. The performance of the models is evaluated on the test set. CEs (upper row), as well as BSs and AUC values (bottom row), are shown across targets for all HP tuning strategies. Results are averaged over ten model repetitions, except for the deep ensemble models, for which five model repeats were computed. For each performance metric and target, an asterisk marks the best model and any those that are statistically indistinguishable from it. Statistical significance was determined in a t-test ($p<$ 0.05)

*Calibration of model selection strategies across targets.* Fig. 5 illustrates the results of the model selection study across all targets and HP metrics. The numerical results can be found in Section A. Figure 5 shows comparable patterns in the ECE and ACE results across targets and HP metrics, allowing us to analyze them collectively as CEs throughout this section. Note that the ACE was slightly smaller than the ECE for all models, as detected in the numerical results in Section A in the Appendix. A reason for this observation could be the high variance of the mean predictions in less populated bins, contributing considerably more to the ECE than other bins and leading to an overestimation of the CE. In summary, models tuned using the BCE loss, and ACE performed better in terms of CE than those tuned on the ACC or AUC values. More specifically, the test set performance of the individual models on the CYP3A4 dataset shows that the model optimizing the ACE and BCE on a validation set results in the best CEs, as illustrated in the first row of Fig. 5. In contrast, optimization based on AUC values leads to the highest CEs. The overall trends detected in the analysis of the CYP3A4 dataset could be also observed with the other two targets. In general, optimizing the HPs with regard to BCE loss or ACE resulted in models that exhibited smaller CEs than optimization based on ACC or AUC. In more detail, HP optimization using the ACE was favorable for the MAO-A dataset, while for the hERG dataset, optimization with the BCE loss led to the significantly smallest CEs.

Since both CEs are improper scores and can give good results for inaccurate models, it is advisable to also consider proper scores when analyzing model calibration. The bottom row of Fig. 5 shows the results of different HP metrics across the three datasets in terms of the proper score BS and the AUC. The BS summarizes the performance of both the calibration of the model and its ability to rank predictions correctly, while the AUC scores solely measure the ranking abilities of the models. Again, the models optimized based on BCE loss and ACE yielded the best BS and AUC values. For targets CYP3A4 and hERG, choosing the BCE loss as HP metric lead to the significantly best BS and AUC scores. Model trained on the MAO-A target, on the other hand, achieved the best BS and AUC results when optimizing for ACE.

Therefore, the BSs and the AUC values confirm the outcomes obtained in the CE analysis, favoring HP selection strategies based on BCE loss or ACE rather than ACC or AUC. This observation could be explained by reduced model overfitting when choosing ACE or BCE loss as HP metric. This assumption is supported by the results of an additional experiment, which investigated the model performance on the training set compared to the test set to demonstrate the overfitting behavior of the models. The study results are displayed in Section B.2 in the Appendix. The experiment showed that the models with HPs optimizing the AUC or the ACC perform best on the training set in terms of AUC, BS, and ACE across all targets. However, when looking at the test performance, the models with HPs optimizing BCE and ACE obtained the best results. The good performance on the training set and comparatively poorer results on the test set imply a more pronounced overfitting of the models with HPs optimizing the ACC or AUC. The reduced overfitting of models tuned using the BCE loss or ACE could be the result of the regularizing effect of the calibration term which contributes to these scores, as shown in Appendix E.

Since ACC and AUC scores do not account for probability calibration, models tuned to optimize these scores on the validation datasets perform worse on the test set.

**Fig. 6:**
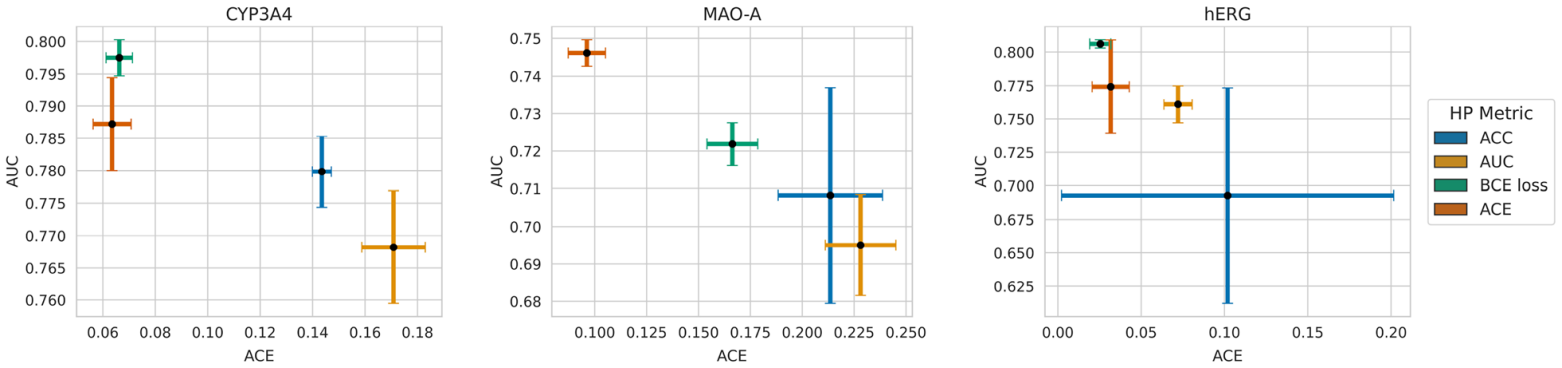
ACE vs AUC across model selection strategies. The ACE of models trained using different HP metrics is plotted against their AUC scores for each target. The performance of the models is evaluated on the test set. Points represent the average values, and error bars indicate the standard deviation based on 10 model repetitions

*ACE vs AUC across model selection strategies.* Fig. 6 illustrates the performance of the models selection strategies in terms of AUC versus AUC scores. This allows for identifying the most suitable HP metric for each situation, enabling the user to select a model that delivers optimal test performance for a specific aspect (calibration, ranking abilities, or any custom-weighted combination thereof). Not that, while there is, in general, a trade-off between calibration and AUC, there is a clear Pareto dominance observed for MAO-A and hERG. Figure 6 shows that ACE as a metric clearly dominates every other HP metric in the case of the target MAO-A, while for hERG using the BCE loss leads to the best-performing model. In the case of CYP3A4, a slight trade-off can be detected since the BCE loss is better in terms of AUC and ACE in terms of ACE. However, the differences in ACE between the model tuned with these two HP metrics are not statistically significant.

These results identify BCE loss as the best choice for HP optimization with regard to BCE loss or AUC. Surprisingly, models tuned to optimize AUC value on a validation dataset also showed worse AUC performance on the test set with HPs optimizing BCE loss or ACE. This observation could result from increased overfitting of the models tuned with metrics that do not consider the calibration error, as reported in the previous section. Due to these results, we will focus on models with HPs that minimize BCE loss in the subsequent sections of this paper. Furthermore, these models achieved the best BS in two targets and the second-best BS for the MAO-A dataset.

### Model calibration study

We compared two popular uncertainty estimation methods and one common probability calibration approach, including the post hoc method Platt scaling and the train-time uncertainty estimation techniques deep ensembles, MC dropout and HBLL. For the sake of a clear and straightforward comparison, we only considered models with HPs selected to minimize the BCE loss in this probability calibration study. The results of models optimizing other HP metrics are listed in Section A in the Appendix.

**Fig. 7:**
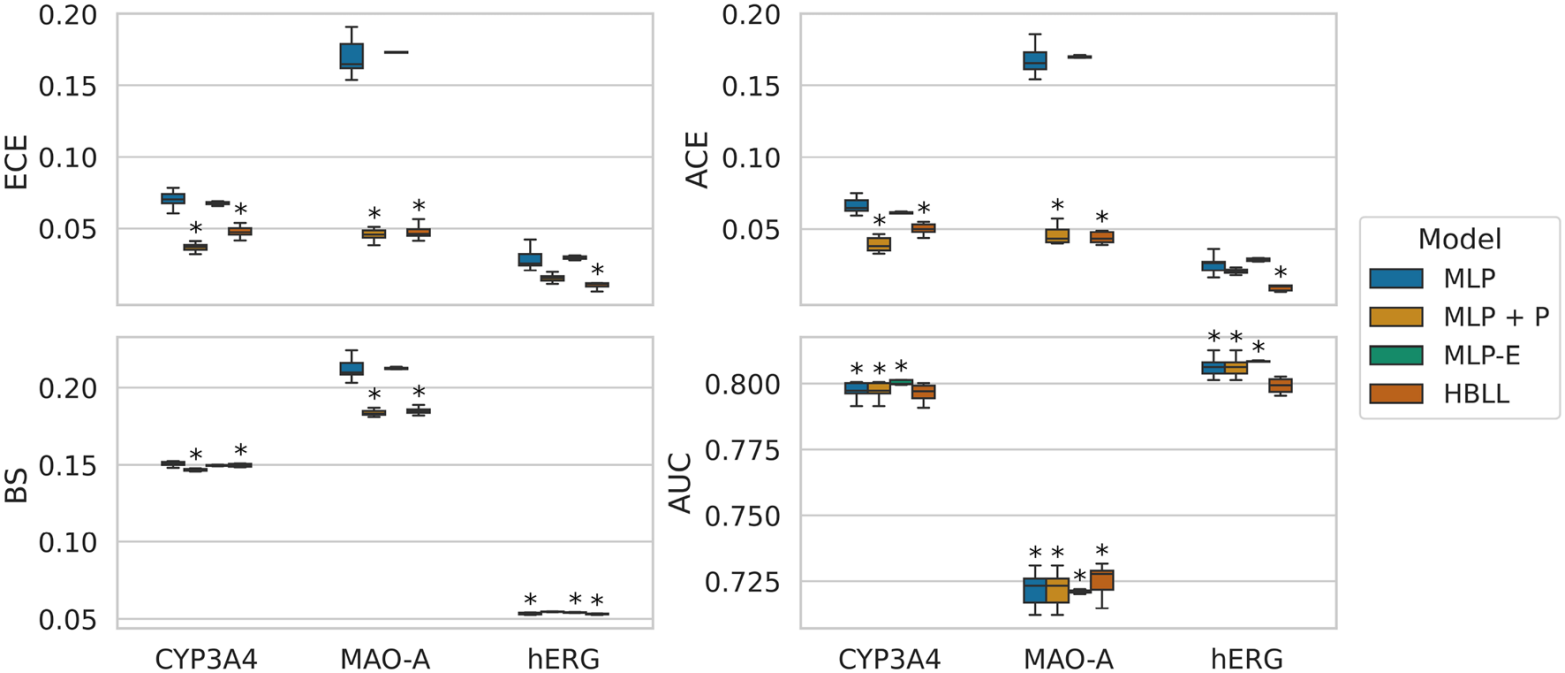
Results of the model calibration study for all targets. The performance of the models is evaluated on the test set. CEs (upper row), as well as BSs and AUC values (bottom row), are shown across targets for all model architectures. Results are averaged over ten model repetitions, except for the deep ensemble models, for which five model repeats were computed. For each performance metric and target, an asterisk marks the best model and any those that are statistically indistinguishable from it. Statistical significance was determined in a t-test ($p<$ 0.05)

*Calibration of model architectures across targets.* Fig. 7 depicts the performance for every target across the model architectures. The MLP-D models performed much worse than the other architectures, and for the sake of a clearer comparison, these results are excluded from Fig. 7. The numerical results of all models, including MLP-D, can be accessed in Section A in the Appendix.

Both calibration errors considered in this study lead to similar results regarding the performance of different model architectures. Besides MC dropout (MLP-D), all models achieve lower CEs than the baseline model for CYP3A4 and MAO-A. However, only MLP + P and HBLL achieve a statistically significant improvement. For hERG, only HBLL significantly improved the calibration errors compared to the baseline MLP. Similar to the CEs results, the analysis of the proper score BS showed that MLP + P and HBLL significantly outperformed other approaches for CYP3A4 and MAO-A. For hERG, no significant difference could be determined between baseline MLP, MLP-E and HBLL. Interestingly, these three approaches outperformed MLP-E in terms of BS. The analysis of the AUC scores showed only minor differences below 0.03 between the model architectures across all targets, which were not statistically significant in the majority of cases.

The results of the models using other HP metrics also support the conclusion that all included methods retrieve similar AUC values, as shown in Section A in the Appendix. An exception to this is MLP-D, which performed poorly in some cases. Based on these results, we can conclude that the analyzed methods improve the probability calibration by decreasing the CEs compared to the baseline model while having a limited impact on the accuracy of the ranking abilities. In this context, interesting findings were reported by Roth and Bajorath, who investigated the relationship between accuracy and calibration in a variety of classification models [67]. Similar to our conclusions, the authors reported that despite their large differences in the calibrating performance, the models overall produced stable and accurate predictions.

In conclusion, Platt scaling (MLP + P) and HMC Bayesian last layer (HBLL) are most powerful in terms of probability calibration as shown in Fig. 7, with the latter resulting in the best calibration error for the MAO-A dataset and hERG. For the hERG dataset, HBLL performed significantly better than all other approaches. HBLL consistently outperformed all other train-time uncertainty estimation approaches across all performance metrics, except for AUC, where the ensemble model (MLP-E) achieved the best results in two targets. These results show that HBLL reaches the CE performance of state-of-the-art uncertainty quantification and probability calibration methods and is only outperformed in one dataset by Platt scaling. Furthermore, common train-time uncertainty quantification methods, such as deep ensembles or MC dropout, do not reach the performance of HBLL or Platt scaling in terms of CEs and BS. A possible explanation for this might be that the rather simple MLP architecture leads to a less complex loss landscape and fewer local minima resulting in similar base estimators of the ensemble model. Small neural networks with fewer neurons and fewer layers have comparably fewer parameters. With parameter count *D*, the loss landscape forms a *D* dimensional hypersurface, which becomes more complex and has more local minima as *D* increases. Furthermore, deep ensembles and MC Dropout were reported to lead to less confident predictions, resulting in more uncertain predictions shifted towards 0.5 [68]. Therefore, these methods do not necessarily improve probability calibration. As a consequence, we hypothesize that ensembling techniques are only beneficial if the base estimators are overconfident, and the predictions are pushed towards 0 or 1. However, they are ineffective for underconfident or well-calibrated models and can even impair the quality of the uncertainty estimates. Over-parametrization of models has been identified as one of the factors contributing to poor model calibration [19]. Given that the models used in this study were rather shallow and their calibration properties were prioritized during HP tuning, including the size of the hidden layer, the resulting base estimators might not suffer from overconfidence in the same severity as it is known for larger deep neural networks.

**Fig. 8:**
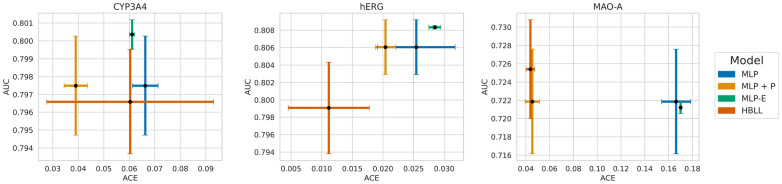
ACE vs AUC across model architectures. The ACE scores of different model architectures are plotted against their AUC scores for each target. The performance of the models is evaluated on the test set. Points represent the average values, and error bars indicate the standard deviation based on 10 model repetitions

*ACE vs AUC across model architectures.* We compared the AUC and ACE of considered model architectures to determine which model is the best choice with regard to different evaluation metrics and to identify potential trade-offs between these metrics. Figure 8 illustrates AUC values plotted against ACE for all model architectures. Again, the MLP-D models were excluded from the plot due to their bad performance. The results for hERG reveal a trade-off between ACE and AUC values across the model architectures. In the plot, the results of the individual models form a Pareto front, with MLP-E performing best in terms of AUC and HBLL in terms of ACE. In this case, it is up to the model user to prioritize either model calibration or ranking abilities to determine a suitable model. The results for the other two models were more noisy, and their pattern was not as clear. For CYP3A4, different models performed best with regard to different evaluation metrics. While MLP + P was best in terms of ACE, MLP-E resulted in the best AUC scores. The analysis of the MAO-A models showed that one single model, namely HBLL, outperformed all others in terms of both metrics (Pareto dominant). However, it is important to note that the AUC differences between many models are not statistically significant, as mentioned in the previous section.

**Fig. 9:**
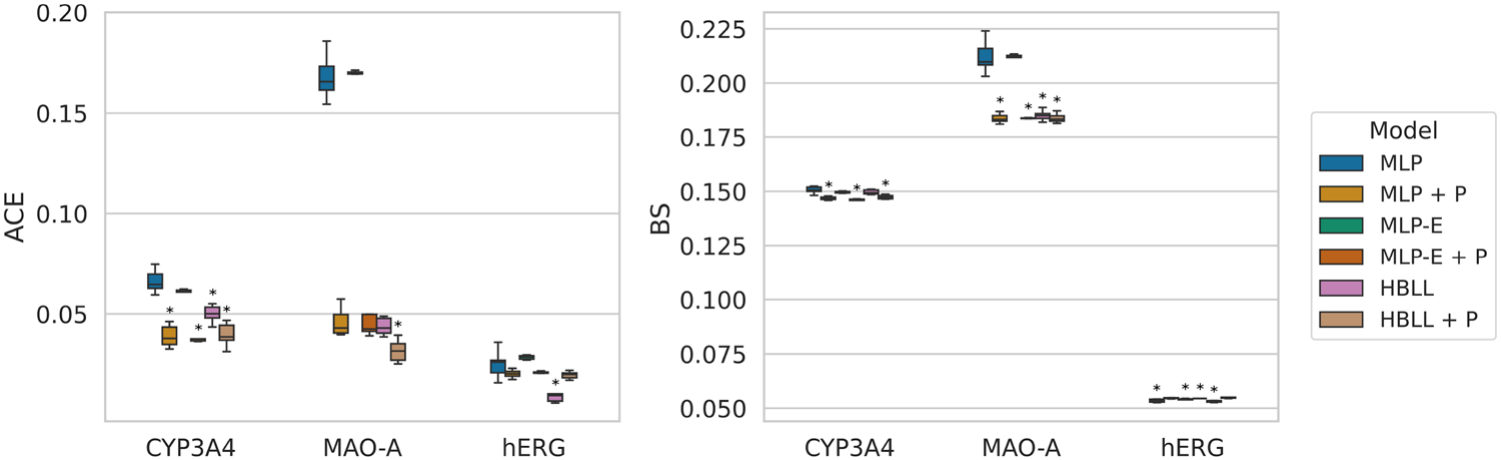
Results of post hoc-calibrated uncertainty quantification methods. The performance of the models is evaluated on the test set. CEs, BSs, and AUC values are shown across targets for selected uncertainty estimation methods and their Platt-scaled counterparts. Results are averaged over ten model repetitions, except for the deep ensemble models, for which five model repeats were computed. For each performance metric, the results of the best model are bold and underlined. All other bold results are statistically indistinguishable from the best result as reported in a t-test ($p<$ 0.05)

*Post hoc calibration of uncertainty quantification methods.* Platt scaling is a post hoc calibration method, which makes it versatile as it can be applied to any model after training. We combined Platt scaling with MLP-E and HBLL to assess if calibrating uncertainty estimates obtained from ensemble modeling or HMC Bayesian last layer enhances model calibration. The results for MLP-E + P and HBLL + P are shown in Fig. 9. Since Platt scaling does not change the ranking of the predictions, the AUC is excluded from this analysis. Furthermore, since the ECE and ACE resulted in similar results in the previous sections, we only report the ACE in Fig. 9 to evaluate the probability calibration of the models. In general, the Platt-scaled models MLP + P, MLP-E + P and HBLL + P outperformed all train-time approaches across all targets. The only model that matched the performance of the Platt-scaled approaches was the HBLL model, which was only significantly outperformed by its Platt-scaled counterpart in terms of ACEs on the MAO-A dataset. The combined methods were reported to significantly improve calibration only in one of the three assays (MAO-A). In this case, the modified HMC Bayesian last layer model HBLL + P achieved the lowest CEs while all other models performed significantly worse. In addition, HBLL + P also generated the smallest BS, with no significant difference to the other Platt-scaled models MLP + P, MLP-E + P and the train-time uncertainty estimation approach HBLL. The results for target CYP3A4 show that, again, all Platt-scaled models performed best, with MLP-E + P resulting in the lowest CEs and BS. The only exception was the HBLL model, which consistently matched the performance of the calibrated models for CYP3A4. The HBLL model also resulted in the best performance of the hERG model, significantly outperforming Platt-scaled models in terms of CEs.

Interestingly, Platt scaling of the ensemble model led to improved CEs across all targets, while the difference between the HBLL and HBLL + P models was much smaller. In some cases, Platt scaling of the HBLL models did not lead to any improvements at all. A possible explanation for this difference between the two uncertainty quantification methods could be that tuning the single HP needed for HBLL model generation already has a calibrating effect. Hence, HBLL models are already well-calibrated and do not need an additional calibration step. As reported in previous sections of this paper, ensembling failed to produce predictions with lower CEs than the baseline in most cases. However, the CEs improved after applying the Platt scaling step.

## Conclusion

In this paper, we provided a systematic study that compared various model selection strategies as well as uncertainty estimation and probability calibration strategies, to achieve well-calibrated models using bioactivity data of three targets extracted from the ChEMBL database [48]. First, we reported that the selection of the metric used for HP tuning substantially affects model performance. We observed that using metrics that took into account the probability calibration of the model, not only resulted in smaller calibration errors but also in improved AUC scores. Second, we compared the baseline model to the common probability calibration method Platt scaling, as well as the train-time uncertainty quantification techniques deep ensembles and MC dropout. In addition, we investigated the calibration performance of the baseline model combined with a Bayesian logistic regression taking as input the output of the penultimate layer, which we called HMC Bayesian last layer (HBLL). A Hamiltonian Monte Carlo (HMC) sampler was used to retrieve samples from the parameter posterior. The results showed that HBLL was the only train-time uncertainty quantification approach that successfully improved the probability calibration over the baseline and could match or outperform other, common uncertainty estimation approaches. Furthermore, the HBLL approach is a good compromise because of its reduced computational complexity compared to the full Bayesian treatment of the weights. Surprisingly, other train-time uncertainty quantification methods failed to produce predictions with smaller calibration errors than the baseline, which might be a result of the previously reported inability of these approaches to calibrate non-overconfident models [68]. In the last step of our analysis, we examined whether applying a post hoc calibrator to different uncertainty estimation methods improved model performance. To do so, we used Platt scaling by fitting a logistic regression to the logits of the deep ensemble and the HBLL model predictions. Interestingly, Platt scaling did not always improve model calibration. In general, CEs of the calibrated models were smaller when the train-time uncertainty estimation model could not improve probability calibration, which was often the case for the results of the ensemble model. The HBLL model already exhibited small CEs before the post hoc calibrating step. Platt scaling of these models produced smaller CEs only in one out of three targets, and it failed to retrieve significantly better BS for all three datasets. In this study, we applied different HP optimization strategies and uncertainty estimation methods to shallow small neural networks. The size of the models was carefully tuned, and shallow neural networks were chosen after concluding that deeper networks did not improve model performance. At the same time, we acknowledge that the small size of the considered models is a limitation of our work since it is uncertain if our findings are also valid for more sophisticated chemical descriptors and larger neural networks. While evaluating such approaches was beyond the scope of the current study, it should be explored in future research. In the framework of the drug discovery process, our work provides important insight into how to achieve reliable uncertainty estimates, facilitating well-informed decision-making and a resource- and time-efficient pipeline for the development of new therapeutic agents.

## Figures and Tables

**Algorithm 1:**
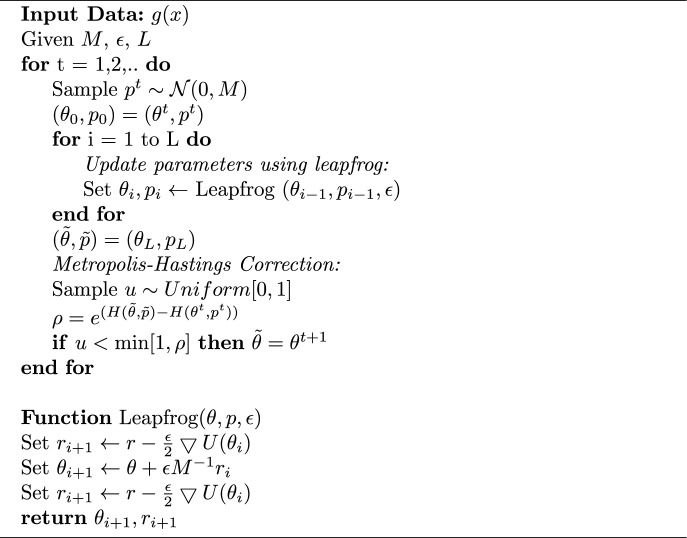
Bayesian Last Layer with Hamiltonian Monte Carlo

## Data Availability

In this study, we use publicly available data sources, which we cite with the corresponding versions in section 2.1. The cleaned ChEMBL data can be downloaded from https://doi.org/10.5281/zenodo.12663462. No datasets were generated or analysed during the current study.

## Appendix A probability calibration study

See Tables 2, 3, 4 and 5.

**Table 2:** Results of the probability calibration study for all targets. The performance of the models is evaluated on the test set. The model HPs were tuned to optimize the AUC score on a validation set. CEs, BSs and AUC values are shown across targets for all probability calibration methods. Results are averaged over 10 model repetitions, except for the deep ensemble models, for which 5 model repeats were computed

HP-Metric: AUC					
Target	Model	ECE	ACE	BS	AUC
CYP3A4	MLP	0.1799 ± 0.0111	0.1709 ± 0.0121	0.1951 ± 0.0103	0.7682 ± 0.0087
	MLP + P	0.0415 ± 0.0072	0.0438 ± 0.0068	0.1586 ± 0.0048	0.7682 ± 0.0087
	MLP-D	0.2262 ± 0.0964	0.2262 ± 0.0948	0.2395 ± 0.0539	0.6968 ± 0.0276
	MLP-E	0.1142 ± 0.0017	0.1054 ± 0.0024	0.1602 ± 0.0002	0.7957 ± 0.0015
	MLP-E + P	0.0551 ± 0.0046	0.0542 ± 0.004	0.1503 ± 0.0002	0.7957 ± 0.0015
	HBLL	0.0452 ± 0.0114	0.0448 ± 0.0103	0.1589 ± 0.0046	0.7684 ± 0.0083
	HBLL + P	0.0452 ± 0.0114	0.0448 ± 0.0103	0.1589 ± 0.0046	0.7684 ± 0.0083
MAO-A	MLP	0.2379 ± 0.0185	0.2281 ± 0.017	0.252 ± 0.016	0.695 ± 0.0134
	MLP + P	0.0574 ± 0.0125	0.0619 ± 0.0129	0.1926 ± 0.0058	0.695 ± 0.0134
	MLP-D	0.0902 ± 0.0322	0.0942 ± 0.0337	0.2063 ± 0.0123	0.6562 ± 0.0196
	MLP-E	0.1544 ± 0.0053	0.1437 ± 0.0029	0.2099 ± 0.0016	0.7187 ± 0.0028
	MLP-E + P	0.0643 ± 0.0044	0.0578 ± 0.0101	0.1861 ± 0.0011	0.7187 ± 0.0028
	HBLL	0.0607 ± 0.0148	0.0647 ± 0.0128	0.1959 ± 0.0047	0.6824 ± 0.0139
	HBLL + P	0.0607 ± 0.0148	0.0647 ± 0.0128	0.1959 ± 0.0047	0.6824 ± 0.0139
hERG	MLP	0.0763 ± 0.0061	0.0721 ± 0.0085	0.079 ± 0.0052	0.761 ± 0.0139
	MLP + P	0.0102 ± 0.0024	0.015 ± 0.0023	0.0576 ± 0.0005	0.761 ± 0.0139
	MLP-D	0.3175 ± 0.1577	0.3172 ± 0.1578	0.1875 ± 0.1368	0.6205 ± 0.0526
	MLP-E	0.0539 ± 0.0033	0.0535 ± 0.0031	0.0662 ± 0.0008	0.8088 ± 0.0032
	MLP-E + P	0.0126 ± 0.0007	0.0199 ± 0.0026	0.0559 ± 0.0002	0.8088 ± 0.0032
	HBLL	0.0126 ± 0.0007	0.0199 ± 0.0026	0.0559 ± 0.0002	0.8088 ± 0.0032
	HBLL + P	0.0126 ± 0.0007	0.0199 ± 0.0026	0.0559 ± 0.0002	0.8088 ± 0.0032

**Table 3:** Results of the probability calibration study for all targets. The performance of the models is evaluated on the test set. The model HPs were tuned to optimize the accuracy (ACC) on a validation set. CEs, BSs and AUC values are shown across targets for all model types. Results are averaged over 10 model repetitions, except for the deep ensemble models, for which 5 model repeats were computed

HP-Metric: ACC					
Target	Model	ECE	ACE	BS	AUC
CYP3A4	MLP	0.1488 ± 0.0031	0.1435 ± 0.0036	0.1759 ± 0.0015	0.7798 ± 0.0055
	MLP + P	0.037 ± 0.0053	0.0371 ± 0.004	0.1536 ± 0.0018	0.7798 ± 0.0055
	MLP-D	0.0453 ± 0.0123	0.0457 ± 0.0096	0.1559 ± 0.0021	0.7781 ± 0.0049
	MLP-E	0.1369 ± 0.0031	0.1333 ± 0.0032	0.1703 ± 0.0003	0.7877 ± 0.0008
	MLP-E + P	0.0345 ± 0.0012	0.0342 ± 0.0012	0.1517 ± 0.0001	0.7877 ± 0.0008
	HBLL	0.0373 ± 0.0075	0.046 ± 0.0051	0.158 ± 0.0023	0.7741 ± 0.0067
	HBLL + P	0.0373 ± 0.0075	0.046 ± 0.0051	0.158 ± 0.0023	0.7741 ± 0.0067
MAO-A	MLP	0.2207 ± 0.0232	0.2136 ± 0.0252	0.2391 ± 0.0224	0.7082 ± 0.0287
	MLP + P	0.0544 ± 0.0159	0.0604 ± 0.015	0.188 ± 0.0107	0.7082 ± 0.0287
	MLP-D	0.1247 ± 0.0284	0.1275 ± 0.0256	0.2126 ± 0.0088	0.66 ± 0.0292
	MLP-E	0.139 ± 0.0028	0.1345 ± 0.0023	0.2005 ± 0.0011	0.728 ± 0.0038
	MLP-E + P	0.0568 ± 0.0044	0.0628 ± 0.0059	0.1826 ± 0.001	0.728 ± 0.0038
	HBLL	0.0563 ± 0.0166	0.0638 ± 0.0137	0.1897 ± 0.0078	0.7039 ± 0.0292
	HBLL + P	0.0563 ± 0.0166	0.0638 ± 0.0137	0.1897 ± 0.0078	0.7039 ± 0.0292
hERG	MLP	0.1028 ± 0.0991	0.1019 ± 0.0997	0.0792 ± 0.0324	0.6928 ± 0.0806
	MLP + P	0.0091 ± 0.0016	0.0205 ± 0.0045	0.0591 ± 0.0024	0.6928 ± 0.0806
	MLP-D	0.2654 ± 0.0859	0.2645 ± 0.0866	0.138 ± 0.0486	0.6281 ± 0.078
	MLP-E	0.0851 ± 0.0135	0.0766 ± 0.0127	0.0626 ± 0.0023	0.741 ± 0.0064
	MLP-E + P	0.0089 ± 0.0016	0.0217 ± 0.003	0.0566 ± 0.0003	0.741 ± 0.0064
	HBLL	0.0302 ± 0.0128	0.0304 ± 0.0128	0.0595 ± 0.0036	0.6917 ± 0.0835
	HBLL + P	0.0302 ± 0.0128	0.0304 ± 0.0128	0.0595 ± 0.0036	0.6917 ± 0.0835

**Table 4:** Results of the probability calibration study for all targets. The performance of the models is evaluated on the test set. The model HPs were tuned to optimize the BCE loss on a validation set. CEs, BSs and AUC values are shown across targets for all model types. Results are averaged over 10 model repetitions, except for the deep ensemble models, for which 5 model repeats were computed

HP-Metric: BCE loss					
Target	Model	ECE	ACE	BS	AUC
CYP3A4	MLP	0.0698 ± 0.0056	0.0663 ± 0.005	0.1506 ± 0.0014	0.7975 ± 0.0028
	MLP + P	0.0373 ± 0.0036	0.039 ± 0.0046	0.1469 ± 0.0007	0.7975 ± 0.0028
	MLP-D	0.1476 ± 0.0246	0.1523 ± 0.0225	0.1783 ± 0.0095	0.7797 ± 0.0077
	MLP-E	0.0674 ± 0.0012	0.0611 ± 0.001	0.1496 ± 0.0004	0.8004 ± 0.0008
	MLP-E + P	0.0366 ± 0.0018	0.0375 ± 0.001	0.1461 ± 0.0003	0.8004 ± 0.0008
	HBLL	0.0585 ± 0.0333	0.0604 ± 0.0327	0.1521 ± 0.0079	0.7966 ± 0.0029
	HBLL + P	0.0393 ± 0.0063	0.0398 ± 0.0048	0.1473 ± 0.0008	0.7966 ± 0.0029
MAO-A	MLP	0.1696 ± 0.0116	0.1663 ± 0.0122	0.212 ± 0.006	0.7219 ± 0.0057
	MLP + P	0.0473 ± 0.0084	0.0455 ± 0.0061	0.1838 ± 0.0017	0.7219 ± 0.0057
	MLP-D	0.1268 ± 0.0061	0.1259 ± 0.0067	0.2019 ± 0.0043	0.7142 ± 0.0081
	MLP-E	0.1729 ± 0.0016	0.1701 ± 0.0007	0.2124 ± 0.0006	0.7212 ± 0.0007
	MLP-E + P	0.0428 ± 0.0011	0.0446 ± 0.0044	0.1838 ± 0.0002	0.7212 ± 0.0007
	HBLL	0.0465 ± 0.0061	0.0439 ± 0.0037	0.1851 ± 0.0018	0.7254 ± 0.0054
	HBLL + P	0.0355 ± 0.0061	0.0318 ± 0.0049	0.1838 ± 0.0017	0.7254 ± 0.0054
hERG	MLP	0.0289 ± 0.0079	0.0254 ± 0.0063	0.0541 ± 0.0018	0.8061 ± 0.0031
	MLP + P	0.0148 ± 0.0023	0.0204 ± 0.0017	0.0547 ± 0.0003	0.8061 ± 0.0031
	MLP-D	0.0815 ± 0.0186	0.0792 ± 0.0191	0.0607 ± 0.0043	0.8061 ± 0.0072
	MLP-E	0.0294 ± 0.0012	0.0285 ± 0.001	0.0541 ± 0.0002	0.8083 ± 0.0002
	MLP-E + P	0.0133 ± 0.0006	0.021 ± 0.0004	0.0545 ± 0.0001	0.8083 ± 0.0002
	HBLL	0.0111 ± 0.0037	0.0112 ± 0.0066	0.0534 ± 0.0009	0.7991 ± 0.0053
	HBLL + P	0.0133 ± 0.001	0.02 ± 0.002	0.055 ± 0.0004	0.7991 ± 0.0053

**Table 5:** Results of the probability calibration study for all targets. The performance of the models is evaluated on the test set. CEs, BSs and AUC values are shown across targets for all probability model types. The model HPs were tuned to optimize the ACE on a validation set. Results are averaged over 10 model repetitions, except for the deep ensemble models, for which 5 model repeats were computed

HP-Metric: ACE					
Target	Model	ECE	ACE	BS	AUC
CYP3A4	MLP	0.068 ± 0.0062	0.0635 ± 0.0073	0.1548 ± 0.0039	0.7872 ± 0.0072
	MLP + P	0.0419 ± 0.0118	0.0429 ± 0.0119	0.1522 ± 0.0031	0.7872 ± 0.0072
	MLP-D	0.2551 ± 0.0158	0.2542 ± 0.0142	0.2437 ± 0.0075	0.6717 ± 0.0267
	MLP-E	0.0457 ± 0.0026	0.0415 ± 0.0007	0.1451 ± 0.0004	0.8043 ± 0.0007
	MLP-E + P	0.0423 ± 0.0035	0.0435 ± 0.0037	0.1451 ± 0.0004	0.8043 ± 0.0007
	HBLL	0.0461 ± 0.0062	0.047 ± 0.0047	0.1536 ± 0.003	0.7872 ± 0.0078
	HBLL + P	0.0461 ± 0.0062	0.047 ± 0.0047	0.1536 ± 0.003	0.7872 ± 0.0078
MAO-A	MLP	0.0999 ± 0.0076	0.0962 ± 0.009	0.1808 ± 0.0021	0.7461 ± 0.0035
	MLP + P	0.0642 ± 0.0044	0.0677 ± 0.0069	0.1744 ± 0.0011	0.7461 ± 0.0035
	MLP-D	0.0671 ± 0.0064	0.0677 ± 0.0098	0.1771 ± 0.0018	0.7396 ± 0.0051
	MLP-E	0.085 ± 0.0025	0.0921 ± 0.0012	0.1797 ± 0.0004	0.7469 ± 0.0007
	MLP-E + P	0.0713 ± 0.0027	0.0703 ± 0.0015	0.1743 ± 0.0002	0.7469 ± 0.0007
	HBLL	0.0638 ± 0.006	0.0848 ± 0.0073	0.1786 ± 0.0017	0.746 ± 0.0035
	HBLL + P	0.0638 ± 0.006	0.0848 ± 0.0073	0.1786 ± 0.0017	0.746 ± 0.0035
hERG	MLP	0.0328 ± 0.0109	0.0317 ± 0.0111	0.0586 ± 0.0033	0.7742 ± 0.0348
	MLP + P	0.0112 ± 0.0051	0.0166 ± 0.0046	0.0569 ± 0.0015	0.7742 ± 0.0348
	MLP-D	0.4349 ± 0.1518	0.4343 ± 0.1531	0.2737 ± 0.1126	0.5771 ± 0.0763
	MLP-E	0.0157 ± 0.002	0.0165 ± 0.0031	0.0533 ± 0.0003	0.8154 ± 0.0024
	MLP-E + P	0.012 ± 0.0013	0.0195 ± 0.0014	0.0542 ± 0.0002	0.8154 ± 0.0024
	HBLL	0.0188 ± 0.0083	0.0224 ± 0.0096	0.0573 ± 0.0027	0.7797 ± 0.0268
	HBLL + P	0.0188 ± 0.0083	0.0224 ± 0.0096	0.0573 ± 0.0027	0.7797 ± 0.0268

## Appendix B: Additional experiments

### B.1 Performance of deeper MLPs

See Table 6.

**Table 6:** BCE-Loss and AUC achieved on the validation dataset.Models with different numbers of layers are compared. The average and standard deviation of 10 model repeats are shown

Nr. of Layers	Validation Loss	Validation AUC
1	0.2956 ± 0.0014	0.9191 ± 0.0009
2	0.2932 ± 0.0043	0.9180 ± 0.0021
3	0.3051 ± 0.0032	0.9118 ± 0.0005
4	0.3151 ± 0.0062	0.9180 ± 0.0024

### B.2 Model Performance on Training vs. Test set

See Table 7.

**Table 7:** Model performance on the training vs test set for models trained with the CYP3A4, MAO-A, and hERG datasets. Models trained with different HP metrics (ACC, AUC, BCE, ACE) are shown. Model performance is reported in terms of AUC, BS, and ACE. Averages and standard deviations of 10 model repeats are reported

HP Metric	AUC		BS		ACE	
	Train	Test	Train	Test	Train	Test
CYP3A4						
ACC	0.9999 ± 0.0001	0.7798 ± 0.0055	0.0020 ± 0.0001	0.1759 ± 0.0015	0.0048 ± 0.0003	0.1435 ± 0.0036
AUC	0.9999 ± 0.0001	0.7682 ± 0.0087	0.0023 ± 0.0002	0.1951 ± 0.0103	0.0009 ± 0.0002	0.1709 ± 0.0121
BCE	0.9967 ± 0.0002	0.7975 ± 0.0028	0.0299 ± 0.0003	0.1506 ± 0.0014	0.0503 ± 0.0026	0.0663 ± 0.005
ACE	0.9800 ± 0.0030	0.7872 ± 0.0072	0.0516 ± 0.0017	0.1548 ± 0.0039	0.0314 ± 0.003	0.0635 ± 0.0073
MAO-A						
ACC	0.9999 ± 0.0001	0.7082 ± 0.0287	0.0040 ± 0.0001	0.2391 ± 0.0224	0.0006 ± 0.0001	0.2136 ± 0.0252
AUC	0.9999 ± 0.0001	0.6950 ± 0.0134	0.0039 ± 0.0001	0.2520 ± 0.016	0.0002 ± 0.0017	0.2281 ± 0.0134
BCE	0.9996 ± 0.0001	0.7219 ± 0.0057	0.0078 ± 0.0002	0.2120 $\pm 0.006$	0.0031 0.0002	0.1663 ± 0.0122
ACE	0.9961 ± 0.0002	0.7461 ± 0.0035	0.0299 ± 0.0004	0.1808 ± 0.0021	0.0486 ± 0.0007	0.0962 ± 0.0009
hERG						
ACC	0.9999 ± 0.0001	0.6928 ± 0.0134	0.0009 ± 0.0004	0.0792 ± 0.0324	0.0001 ± 0.0008	0.1019 ± 0.0997
AUC	0.9999 ± 0.0001	0.7610 ± 0.0139	0.0012 ± 0.0002	0.0790 ± 0.0052	0.0001 ± 0.0001	0.0721 ± 0.0085
BCE	0.9994 ± 0.0001	0.8061 ± 0.0031	0.0055 ± 0.0001	0.0541 ± 0.0018	0.0027 ± 0.0002	0.0254 ± 0.0063
ACE	0.9409 ± 0.001	0.7742 ± 0.0348	0.0392 ± 0.0005	0.0586 ± 0.0033	0.0070 ± 0.0005	0.0586 ± 0.0111

## Appendix C: Hyperparameter tuning

See Tables 8 and 9.

**Table 8:** Overview of the parameter space screened during HP tuning of the baseline MLP model. The HP were tuned for each dataset using four different HP metrics. The assess the performance on the validation set, the average of ten model repetitions was obtained. The overview presents the range of HP explored during the tuning process

Model	Hyperparameter	Explored Space
Baseline MLP	Learning rate	{1e-5, 1r-4, 1e-3, 1e-2, 0.1}
	Hidden size	{5, 10, 20, 30, 40, 50, 60, 70, 80, 90, 100, 110, 120, 130}
	Weight decay	{1e-5, 1r-4, 1e-3, 1e-2, 0.1}
	Dropout	{0.3, 0.4, 0.5, 0.6, 0.7, 0.8, 0.9}

**Table 9:** Overview of the parameter space screened to tune the prior of the parameters from a Bayesian Logistic regression, which was used as the last layer in the HBLL models. More precisely, the precision of the Gaussian prior of the model parameters was optimized using the validation set

Model	Hyperparameter	Explored Space
HBLL	Precision of the	{100, 150, 200, 250, 300, 400, 600,
	Gaussian prior	800, 1000, 1200, 1400, 1600, 1800, 2000}

## Appendix D: Computation time of models

See Table 10.

**Table 10:** Time required for models included in the study. The left column lists the time needed for training ($T_{train}$), and the right column the time needed for inference ($T_{inference}$). The meanings of the abbreviations used in the table are provided below. Typical training times for models ($T_{train}$) used in this study are listed in the last column

Model	$T_{train}$	$T_{inference}$	Typical Training Time
MLP	$n_{m} * (T_{f} + T_{b})$	$T_{f}$	$\sim$ 7:30 min
MLP-D	$n_{m} * (T_{f} + T_{b})$	$n_{f} * T_{f}$	$\sim$ 7:30 min
MLP-E	$n_{e} * n_{m} * (T_{f} + T_{b})$	$n_{e} *T_{f}$	$\sim$ 188 min
HBLL	$n_{m} * (T_{f} + T_{b}) + L * n_{s} * (T_{flast} + T_{s})$	$n_{s} * T_{f}$	$\sim$ 175 min

*1) Required time* *T* *for...*

$T_{f}$: *one forward pass,* $T_{b}$: *one backpropagation step,*

$T_{s}$: *one HMC step,* $T_{flast}$: *forward pass of last layer *

*2) Number of..*

$n_{m}$: *mini-batches*, $n_{f}$: *forward passes in MLP-D*, $n_{e}$: *base estimators*

*in MLP-E*, $n_{s}$: *samples in HMC*, *L*: *simulation steps in HMC*

## Appendix E: Risk decomposition for proper losses: calibration and refinement

In the following section, we give some necessary definitions to present the decomposition property of the expected proper loss to calibration error and refinement. In our paper we use the word calibration and calibration error in the specific sense implied by this decomposition.

To formally define calibration, first we need to define a proper loss: a proper score which is negatively oriented, meaning smaller values of the score indicates better agreement with the ground truth distribution of the outcomes. We give all definitions for the case of binary classification for simplicity.

### Definition E.1

*(Proper loss)* A loss function $\mathcal {L}: \Delta ^2, \{0,1\} \rightarrow \mathbb {R}$ is a proper loss function if and only if

$$\begin{aligned} \mathbb {E}_{\omega \sim Q}[\mathcal {L}(Q,\omega )] \le \mathbb {E}_{\omega \sim Q}[\mathcal {L}(P,\omega )] \end{aligned}$$

for all distribution *P*, where *Q* is the ground truth distribution over the outcomes, and $\Delta ^2$ is the 2 dimensional probability simplex, that is $\Delta ^2 \subset \mathbb {R}$ where $[x, 1-x] \in \Delta ^2$.

We will use the property that every proper loss can be written in a form of a Bregman divergence [69].

### Definition E.2

*(Bregman divegence)* Let $F: \Omega \rightarrow \mathbb {R}$ be a continuously differentiable strictly convex function defined on a convex set $\Omega$. The Bregman divergence associated with *F* is defined as

$$\begin{aligned} D_F(p,q) = F(p) - F(q) - \left<\nabla F(q),p-q\right>, \end{aligned}$$

where $$p,q \in \Omega$$.

For example the Brier loss can be derived using $\Omega = \Delta ^2$ and $F(p) = ||p||^2$. Using the definition of Bregman divergences:

5 $$\begin{aligned} D_F(p,q)&= ||p||^2 - ||q||^2 - 2q^{\top }\left( p-q\right) \end{aligned}$$

6 $$\begin{aligned}&= ||p||^2 - ||q||^2 - 2q^{\top }p + 2||q||^2 \end{aligned}$$

7 $$\begin{aligned}&= ||p||^2 + ||q||^2 - 2q^{\top }p \end{aligned}$$

8 $$\begin{aligned}&= ||p - q||^2 \end{aligned}$$

We call the expectation of the loss over the data distribution the Risk. The definition for binary classification is the following.

### Definition E.3

*(Risk)* Let $\mathcal {L}: \Delta ^2, \{0,1\} \rightarrow \mathbb {R}$ be a loss function, and $f: \mathcal {X} \rightarrow \Delta ^2$ a classifier mapping from the input space to the probability space. We define the following expected loss as the Risk associated to the classifier:

$$\begin{aligned} \mathcal {R} = \mathbb {E}_{X,Y \sim P(X,Y)} \left[ \mathcal {L}(f(X), Y) \right] . \end{aligned}$$

In the following we will omit the dataset distribution *P*(*X*, *Y*) from the subscript of the expectations to improve readability.

Using the properties of Bregman divergences, the Risk associated with a proper loss can be decomposed in the following form [69, 70]:

$$\underbrace{\mathbb {E}[D_{F}(f(x),y)]}_{\text {Risk}} = \underbrace{\mathbb {E}[D_{F}(\mathbb {E}[y|f(x)], f(x))]}_{\text {Calibration error}} + \underbrace{\mathbb {E}[-F(\mathbb {E}[y|f(x)]]}_{\text {Refinement}}$$

A crucial element of the above expression is the conditional expectation $\mathbb {E}[y|f(x)]$. This intuitively represents the expected fraction of positive outcomes, given the prediction is *f*(*X*). This expectation is not available in a closed form. Histogram estimate is used by ACE and ECE to estimate this quantity by counting the positive outcomes for different $\tau _i \le f(x) < \tau _{i+1}$ bins:

$$\begin{aligned} \mathbb {E}[y|f(x)] \approx \bar{o}_i = \frac{\sum _i y_i I_{\tau _1< f(x_i)< \tau _2}}{\sum _i I_{\tau _1< f(x_i) < \tau _2}}, \end{aligned}$$

where $I_E$ is an indicator 1 if *E* logical expression is true and 0 otherwise. Intuitively the above expression gives the ratio of positive examples in the *i*th bin.

The loss between this estimate and the mean probabilistic prediction *f*(*x*) within the bin is the calibration error. Most often $L_1$ loss (absolute distance) is used in the literature, resulting in the ACE and ECE estimates.

It is easy to show that the $L_2$ calibration error directly corresponds to the calibration part of the Brier score [69]:

$$\begin{aligned} \mathbb {E}[||f(x)-y)||^2] = \mathbb {E}[||\mathbb {E}[y|f(x)]-f(x)||^2]-\mathbb {E}[||\mathbb {E}[y|f(x)]||^2] \end{aligned}$$

While using histogram estimate is the most often used solution, it is not the only possibility. Recently a new class of differentiable estimates was published based on Dirichlet kernel density estimator [71].

## Appendix F: Training of and prediction with multilayer perceptrons (MLP)

This section aims to give the mathematical background for training and predicting with multilayer perceptrons (MLPs). For more details, we refer to [72]. MLPs are feedforward neural network models that consist of multiple layers of interconnected nodes that apply nonlinear functions to a linear combination of the inputs.

For the input variables $X:= \{{\varvec{x_1}},..., {\varvec{x_M}}\}$, the fist layer (1) of hidden size *M* is constructed by taking the linear combinations of these inputs weighted by the weights $w_{ji}^{(1)}$ and adding biases $b_{j0}^{(1)}$, with $j = 1, 2,...M$:

9 $$\begin{aligned} z_{k}^{(1)} = \sum _{i=1}^{D}w_{ji}^{(1)}x_{i} + b_{j}^{(1)}. \end{aligned}$$

Subsequently, a nonlinear activation function *h*(.) is applied to retrieve the output activations:

10 $$\begin{aligned} a_{k}^{(1)} = h(z_{k}). \end{aligned}$$

In our work, we use Rectified linear unit (ReLU) functions as activation functions. The retrieved output activations can be again transformed by additional layers, following the same concept. These layers take the activated output of the previous layer as input and apply a nonlinear activation function to the linear combinations of input, and the model parameters $\theta$ (weights and biases).

The MLP baseline models used in this study consist of one layer as described in Eq. F6, plus a second linear layer of size 1. Subsequently, these scores are transformed to probability-like output using a logistic sigmoid function $\sigma (.)$. An illustration of the architecture of the baseline models can be found in Fig. 2.

### F.1 Forward pass

A forward pass through the MLP refers to passing input data through the network’s layers to compute the probabilistic output using the current model parameters $\theta$.

The output *y* of the baseline MLP model used in this work can be obtained with

11 $$\begin{aligned} y({\varvec{x}}, \theta ) = \sigma ( \sum ^{M}_{j = 1}w^{(2)}_{j}h( \sum ^{D}_{i = 1}w^{(1)}_{ji} + b_{j}^{(1)} ) + b^{(1)}). \end{aligned}$$

During inference, forward passes are performed to process input data through the network and generate predictions. Furthermore, forward passes are an integral part of the neural network training process, used to compute the training loss, which is then employed to adjust the model parameters through backpropagation.

### F.2 Training by error backpropagation

The backpropagation step is essential for neural networks to learn from the data. The goal is to minimize the error between the predicted output and the target value by adjusting the parameters of the model. This error can be obtained by using a loss function, such as the BCE loss, which is commonly used for classification tasks and was also used for model training in this work. During model training, forward passes are used to obtain predictions for the training data and calculate the error. Subsequently, the error is used in the backpropagation step to modify the model’s parameters. Backpropagation relies on the chain rule to compute the gradients of the loss function with respect to each parameter. Thus, using Loss $\mathcal {L}$, for a single weight in layer *l*, we can compute the gradient as

12 $$\begin{aligned} \frac{\partial \mathcal {L}}{\partial w_{jk}^{(l)}} = \frac{\partial \mathcal {L}}{\partial z_{j}^{(l)}} \frac{\partial z_{j}^{(l)}}{\partial w_{jk}^{(l)}} \end{aligned}$$

in which

13 $$\begin{aligned} z_{j}^{(l)} = \sum _{k = 1}^{M}w_{jk}^{(l)}a_{k}^{l-1} + b_{j}^{l}. \end{aligned}$$

We now can define the error $\delta _{j}^{l}$ in neuron *j* in layer *l* as

14 $$\begin{aligned} \delta _{i}^{j} = \frac{\partial \mathcal {L}}{\partial z_{j}^{(l)}} \end{aligned}$$

Using the chain rule, the error can be propagated through the network starting from the output layer (backward pass). The error associated with each layer *l* is stored as $\delta ^{l}$, used for efficiently calculating the local gradients for the parameters in the previous layers. Subsequently, the weights ${\varvec{W}}^{l}$ of a layer *l* can be updated with

15 $$\begin{aligned} {\varvec{W}}^{l} ={\varvec{W}}^{l} - \eta \frac{\partial \mathcal {L}}{\partial {\varvec{W}}^{l}} \end{aligned}$$

where $\eta$ denotes the stepsize. The same procedure can be used to update the values for the bias. Additional details are available in [72].

## Appendix G: Algorithm: HMC bayesian last layer

We report the algorithm for the HBLL approach. To simulate a smooth trajectory through the negative log space of the unnormalized posterior, the auxiliary momentum variable (*p*) is introduced. Together with the position variables $\theta$, the total energy of the system *H* can be calculated; Hamiltonian function is defined as $H = U(\theta + \frac{1}{2}r_{T}$.

After sampling the position $\theta$ and the momentum *p*, the leapfrog integrator is used to obtain the trajectories to generate samples $t:= \{1,2,...T\}$. At the end of each simulation, a Metropolis-Hastings step is applied to check if the energy was preserved and the sample $\tilde{\theta }$ is accepted. We refer to [46] for more details on HMC.

We introduce $\theta$ as the randomly initialized parameters of the logistic regression and the auxiliary variable (*p*) for the momentum. Let ${\varvec{X}}:= \{{\varvec{x_{0}}}, {\varvec{x_{1}}},...{\varvec{x_{N}}}\}$ be the test data and $g({\varvec{x}})$ be the output of the first layer of the previously trained baseline MLP. *M* is the mass matrix, while stepsize is denoted with $\epsilon$ and the number of steps with *L*.

## Code availability

We only used third-party open-source software cited in the Methods section of this paper. The code is available on GitHub: https://github.com/hannahrosafriesacher/CalibrationStudy.
